# Potential Anti-Cancer Activities and Mechanisms of Costunolide and Dehydrocostuslactone

**DOI:** 10.3390/ijms160510888

**Published:** 2015-05-13

**Authors:** Xuejing Lin, Zhangxiao Peng, Changqing Su

**Affiliations:** Department of Molecular Oncology, Eastern Hepatobiliary Surgical Hospital & National Center of Liver Cancer, Second Military Medical University, Shanghai 200438, China; E-Mails: linxuejinga@163.com (X.L.); pengzhangxiao@126.com (Z.P.)

**Keywords:** costunolide, dehydrocostuslactone, cancer treatment, molecular mechanism

## Abstract

Costunolide (CE) and dehydrocostuslactone (DE) are derived from many species of medicinal plants, such as *Saussurea lappa* Decne and *Laurus nobilis* L. They have been reported for their wide spectrum of biological effects, including anti-inflammatory, anticancer, antiviral, antimicrobial, antifungal, antioxidant, antidiabetic, antiulcer, and anthelmintic activities. In recent years, they have caused extensive interest in researchers due to their potential anti-cancer activities for various types of cancer, and their anti-cancer mechanisms, including causing cell cycle arrest, inducing apoptosis and differentiation, promoting the aggregation of microtubule protein, inhibiting the activity of telomerase, inhibiting metastasis and invasion, reversing multidrug resistance, restraining angiogenesis has been studied. This review will summarize anti-cancer activities and associated molecular mechanisms of these two compounds for the purpose of promoting their research and application.

## 1. Introduction

Medicinal plants have been used to treat cancer for thousands of years in Ancient Egypt, India, China, and Arab world. Statistically, more than 3000 plant species were used for treating cancer worldwide [1]. Plant-derived anticancer agents are an important source of anticancer drug due to more structurally diverse “drug-like” and “biologically friendly” molecular qualities than pure synthetic compounds at random [2]. Currently, four major structural classes of plant-derived agents, namely, the epipodophyllotoxin lignans, the taxane diterpenoids, the vinca alkaloids, and the camptothecin quinoline alkaloid derivatives are used in medicine as single chemical entity compounds [3]. Costunolide (CE) and dehydrocostuslactone (DE), two natural sesquiterpene lactones, present in a number of medicinal plants such as *Saussurea lappa* and *Laurus nobilis*, have caused intense interest in researchers due to their potential anti-cancer activities for various types of cancer, such as, leukemias [4], liver cancer [5,6], breast cancer [7,8], ovarian cancer [9,10], prostatic cancer [11,12], and bladder cancer [13]. However, the molecular mechanisms for the anticancer activities of CE and DE are largely ambiguous. Therefore, in the following sections of this short review, we will provide a brief overview of anti-cancer activities and associated molecular mechanisms of these two compounds.

## 2. General Pharmacology

**Figure 1:**
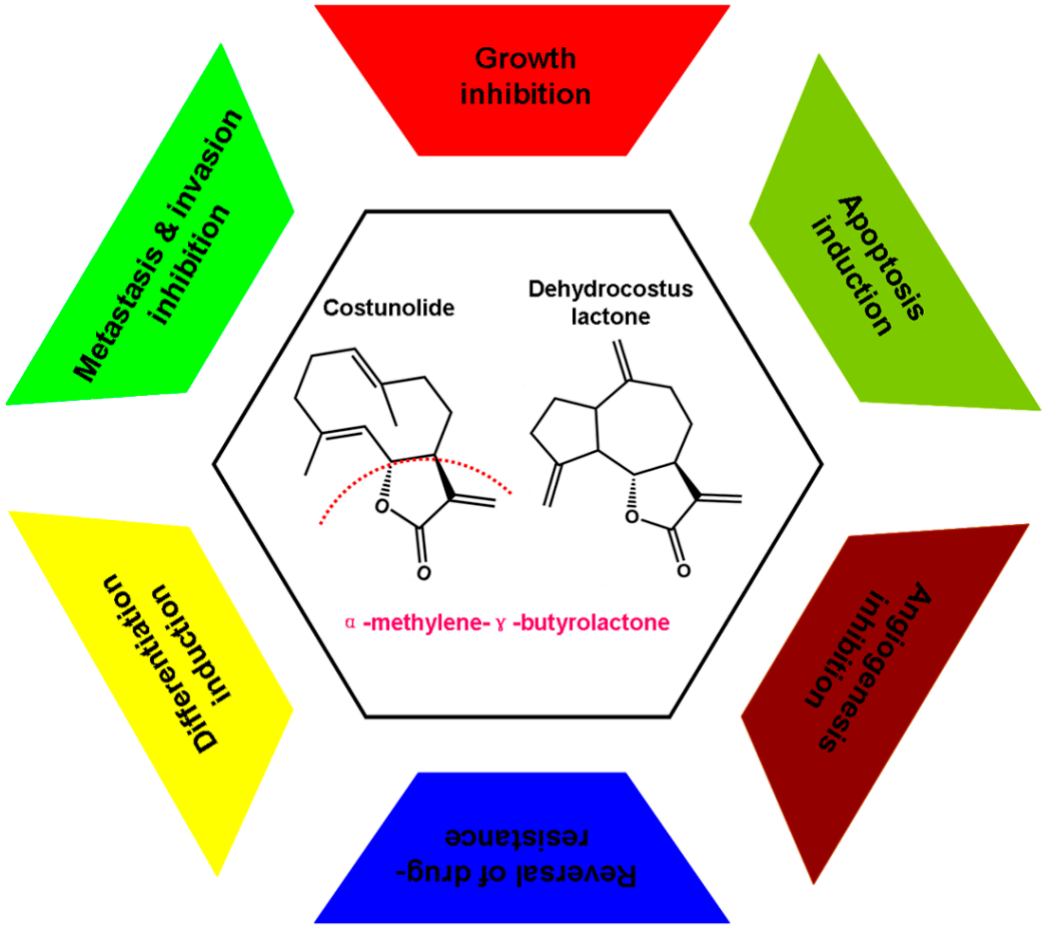
Chemical structure and possible anti-cancer mechanisms of Costunolide (CE) and dehydrocostuslactone (DE). CE (C_15_H_20_O_2_) and DE (C_15_H_18_O_2_) could exert their anti-cancer activities mainly by six pathways, including growth inhibition, cell cycle regulation, apoptosis induction, inhibition of angiogenesis, inhibition of invasion and metastasis, differentiation induction and reversal of drug resistance.

Dried four- to five-year-old roots of *Saussurea lappa*, known as costus root, have a reputation for their usage in traditional medicine systems in India, China, Japan, and Pakistan [14]. CE and DE, two sesquiterpene lactones, are the major chemical and bioactive constituents of *Saussurea lappa* [15]. Much evidence indicates that the α,β-unsaturated carbonyl group in the α-methylene-γ-butyrolactone (Figure 1) moiety of CE and DE may play some pivotal roles through conjugation with mercapto (SH)-groups of target proteins to intervene in some key biological processes in cells [16,17,18,19,20]. Therefore, these two compounds possess various biological activities, including anti-inflammatory [21,22], anticancer [23,24], antiviral [25], antimicrobial [26,27], antifungal [28], antioxidant [29,30], antidiabetic [31], antiulcer [32], and anthelmintic activities [33]. Additionally, they also can lower blood pressure, relieve spasms of smooth muscles, dilate the bronchi, and improve stomach function [34,35].

Currently, clinically available CE and DE-containing drugs, such as, Aplotaxis Carminative Pill, Aucklandiae and Areca Pill, and Compound Ancklandia and Berberine Tablets have been used for treatment of digestive tract diseases with their anti-inflammatory, antimicrobial and stomach function-improving activities.

## 3. Experimental Anti-Cancer Activities and Associated Molecular Mechanisms

Carcinogenesis is a multistep process activated by altered expression of oncogenes and transcriptional factors that are involved in cell proliferation, cell cycle regulation, cell apoptosis, cell differentiation, angiogenesis, cell invasion, and metastasis [36]. Increased angiogenic potential, proliferation, invasion and metastasis capacities, accompanied with uncontrolled cell cycle progression and apoptosis inhibition are the hallmark of cancer. Accordingly, the agents targeting one or more of these processes should be ideal cancer chemopreventive agents [37]. A great number of research results supported that CE and DE can exert their anti-cancer activities by various pathways, mainly manifested in inhibition of cancer cell proliferation [5], induction of cancer cell apoptosis and differentiation [9,38], inhibition of metastasis and invasion [11], reversal of multidrug resistance [39], and inhibition of angiogenesis [40] (Figure 1).

## 4. Inhibition Effect on Cancer Cell Proliferation

### 4.1. Modulation of Cell Cycle Progression

The unlimited growth of cancer cells is closely associated with the uncontrolled cell cycle regulation mechanism. The cell cycle is regulated by a complex network consisting of positive and negative cell cycle regulatory molecules, such as cyclins, cyclin-dependent kinases (Cdks), and Cdk inhibitors [41]. CE and DE treatments have been shown to inhibit the proliferation of various cancer cells, including human hepatocellular carcinoma HA22T/VGH cells [5], ovarian cancer OVCAR3 [7] and SK-OV-3 cells [9], bladder cancer T24 cells [13], breast cancer MDA-MB-231 cells [42], gastric adenocarcinoma SGC-7901 cells [43], soft tissue sarcomas SW-872, SW-982 and TE-671 cells [44].

Most studies indicate that CE and DE inhibit cell cycle progression through an increase of the G2/M phase combined with a depletion of the G0/G1 phase in various cancer cells. It is observed that CE arrests bladder cancer T24 cell cycle at G2/M phase, and the proportion at the G2/M phase is increased from 13.78% ± 1.26% in the control group to 25.64% ± 2.16% and 41.32% ± 2.66% in the CE-treated group at the dose of 25 and 50 μM for 24 h, respectively [13]. CE can reduce the expression of cyclin B1 and Cdc2, and increase the expression of p21^AWF1^ and the binding Cdc2-p21^AWF1^, independent of p53 pathway in p53-mutant MDA-MB-231 cells, resulting in G2/M arrest [42]. In addition, CE can markedly up-regulate the expression of phosphorylated Chk2 (Thr 68), phosphorylated Cdc25c (Ser 216), phosphorylated Cdk1 (Tyr 15) and cyclin B1 in HA22T/VGH cells, which lead to cell arrest at mitosis, not G2 phase [5].

**Figure 2:**
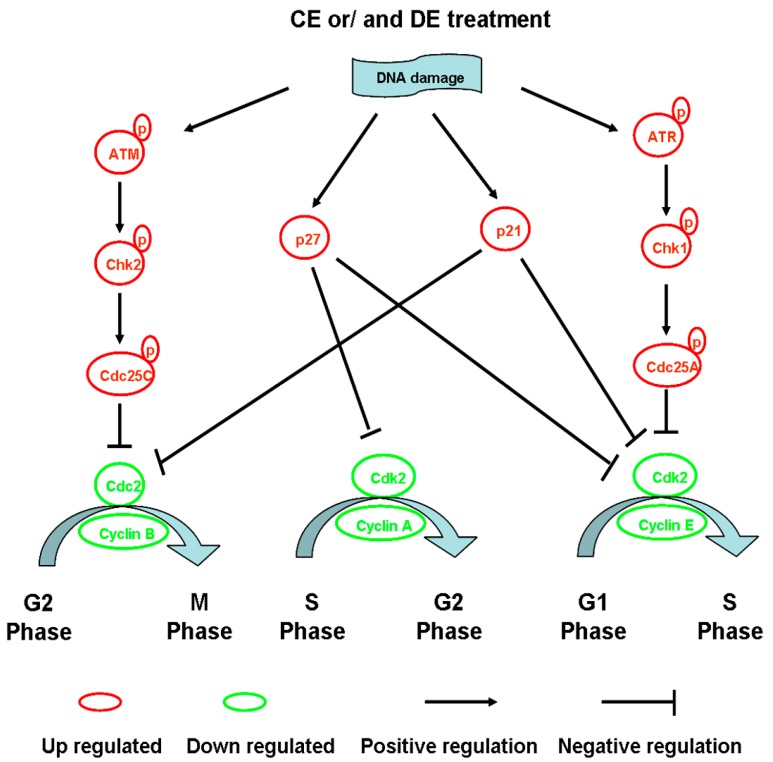
The mechanism of Costunolide (CE) or/and dehydrocostuslactone (DE)-induced cell cycle arrest. CE or/and DE inhibit cell cycle progression mainly through an increase of G2/M phase and S phase. ATM, ataxia telangiectasia mutated; Cdk, cyclin-dependent kinase; ATR, ATM and Rad3-related; Chk, checkpoint kinases; Cdc, cell division cycle protein.

Studies from Choi *et al.* demonstrated that DE induces G2/M phase arrest in human ovarian cancer SK-OV-3 cells through up-regulation of p21, down-regulation of Cdk1 as well as cyclin A and cyclin B, which combine with Cdk1 in controlling the G2/M transition [9]. Lohberge *et al.* reported that DE arrest cells at the G2/M phase and cause a decrease in the expression of Cdk2, Cdc2 (Cdk1) and cyclin B1 in human soft tissue sarcoma cells. In addition, a few studies indicated that DE also induces S and G0/G1 arrest in cancer cells [45]. Kuo and Kretschmer *et al.* investigated that DE blocks *S*-phase progression through Cdk inhibitor up-regulation and cyclin inhibition pathways [23,44]. Moreover, Wang *et al.* reported that DE causes G0/G1 phase arrest by decreasing the expression of cyclinD1 and Cdk2 in human umbilical vein endothelial cell (HUVEC) [46]. Similar results were also reported in human prostate cancer cells [47]. These studies suggested that regulation of cell cycle-associated regulatory factors is one of the mechanisms of CE and DE in prevention and therapeutic intervention of cancer (Figure 2).

Interestingly, many secondary metabolites in plants can modulate the cell cycle progression through the activation of p53/p21/p27 pathway [48,49]. It is well documented that secondary metabolites can act on the redox equilibrium of mammalian cells in *in vitro* experiments [50,51]. Lee *et al.* [52] and Choi *et al.* [9] reported that CE and DE can deplete intracellular thiols and lead to the generation of reactive oxygen species (ROS) in cells, which will induce DNA damage and cancer cell apoptosis. p53 is a well-known tumor suppressor protein and its activation and accumulation in the nucleus in cells are always accompanied by the occurrence of DNA damage in cells. Of the p53 targets, the p21 and p27 Cdk inhibitors are the most investigated genes because of their ability to induce cell cycle arrest. An increase in p21 or p27 can be easily associated with changes in cell cycle arrest, including G0/G1, S or G2/M phase block [53]. Taken together, the activation of p53/p21/p27 is a very common mechanism of anti-cancer activity for secondary metabolites in plants.

### 4.2. Influence of Tubulin Polymerization

Microtubules composed of α- and β-tubulin heterodimers, the main ingredients of the cell cytoskeleton, play a crucial part in diverse cellular functions, such as intracellular transport, metabolism, cell shape, migration, and so on. Therefore, they have long been considered as an important target for cancer therapy [54,55,56]. Currently, taxanes and vinca alkaloids have been used as tubulin-targeting anticancer drugs, and their anti-cancer mechanisms might be associated with restructuring and reorganizing microtubules [57]. It has been reported that CE can selective target detyrosinated tubulin, in turn reduce the frequency of microtentacles and inhibit tumor cell reattachment independent of nuclear factor-kappa B (NF-κB) activation. More interestingly, CE can decrease detyrosinated microtubules without disrupting the overall microtubule network, therefore, it presents a novel anti-cancer activity, low toxicity and therapeutic potential for breast cancer therapy [58]. Studies from Bocca *et al.* demonstrated that CE can exert a dose-dependent antiproliferative activity in the human breast cancer MCF-7 cells as a microtubule-interacting agent. After treatment with 100 nM of CE, the size of cells varies and the microtubules appear as a fine network of dense and unaligned fibers [59]. These studies demonstrated that CE can be related to an interaction with microtubules and inhibits the proliferation of cancer cells.

### 4.3. Inhibition of Telomerase Activity

The human telomerase complexes are comprised of telomerase reverse transcriptase (hTERT), telomerase RNA components (hTR) and other telomerase proteins, however hTERT, a specialized ribonucleoprotein, is the key component of telomerase, which plays an important part in cell proliferation as a protective mechanism against end-replication problems by adding TTAGGG repeats to the telomeres [60,61]. Compared with normal cells, most cancer cells have high telomerase activity, which leads to enduring proliferation of the cancer cells and development of malignant tumors [62,63]. Therefore, telomerase has been considered as a possible target for cancer therapy. In the present study, Choi demonstrated that CE inhibits the growth of breast cancer MCF-7 and MDA-MB-231 cells by down-regulation of hTERT and transcriptional factors c-Myc and Sp1, and inhibition of telomerase activity [64]. In addition, CE induced apoptosis and suppression of hTERT via the receptor-mediated pathway in human leukemia NALM-6 cells [65]. Thus, CE could potentially be used to study the molecular mechanism of inhibition of telomerase, as well as for preclinical and clinical studies aimed at telomerase targeting.

### 4.4. Induction of Cell Apoptosis

**Figure 3:**
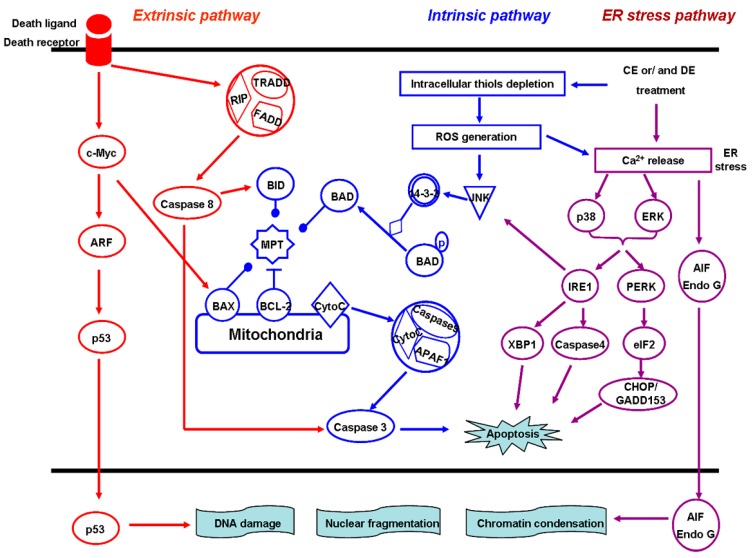
Costunolide (CE) or/and Dehydrocostuslactone (DE)-induced cell apoptosis pathways. CE and/or DE induce cell apoptosis mainly through the mitochondria-dependent “intrinsic” cytochrome C/caspase-9 pathway, the death receptor-mediated “extrinsic” caspase-8 pathway, and the endoplasmic reticulum (ER) stress pathway. The three pathways are linked in that molecules in one pathway can influence the other. ARF, alterative reading frame; TNF receptor-associated death domain (TRADD), TNF receptor-associated death domain; FADD, Fas-associated death domain; RIP, Receptor-interacting protein; BAX, BCL-2 associated X protein; BID, BH3 interacting domain death agonist; BAD, BCL-2 antagonist of cell death; Bcl-2, B-cell lymphoma protein 2; MPT, mitochondrial permeability transition; 14-3-3, Tyrosine 3-monooxygenase/tryptophan; JNK, c-Jun *N*-Terminal Kinase; ERK, extracellular signal regulated kinase; IRE1, inositol-requiring protein 1; PERK, double-stranded RNA-activated protein kinase-like endoplasmic reticulum kinase; AIF, apoptosis-inducing factor; Endo G, endonuclease G; XBP-1, X-box transcription factor-1; eIF-2, eukaryotic translation initiation factor-2.

Apoptosis, also known as programmed cell death, is a ubiquitous and highly regulated mechanism, involving an energy-dependent cascade of molecular events [66]. The morphological features of apoptosis can be characterized as cell shrinkage and convolution, pyknosis and karyorrhexis, intact cell membrane and cytoplasm retained in apoptotic bodies [67]. To date, research indicates that there are at least three CE- and/or DE-induced apoptotic pathways: (i) The mitochondria-dependent “intrinsic” cytochrome C/caspase-9 pathway; (ii) The death receptor-mediated “extrinsic” caspase-8 pathway; and (iii) the endoplasmic reticulum (ER) stress pathway [6,68,69]. The three pathways are linked in that molecules in one pathway can influence the other [67,70] (Figure 3).

### 4.5. The Mitochondria-Dependent Intrinsic Pathway

Non-receptor-mediated stimuli, such as toxins, hypoxia, hyperthermia, viral infections, and the absence of certain growth factors and hormones, can cause an opening of the mitochondrial permeability transition (MPT) pore, and loss of the mitochondrial transmembrane potential, which results in releasing two main groups of normally sequestered pro-apoptotic proteins from the intermembrane space into the cytosol and triggering the intrinsic signaling pathways of apoptosis [71,72,73,74]. The BCL-2 family of proteins, including the anti-apoptotic proteins, BCL-2, BCL-X, BCL-XL, and so on, and the pro-apoptotic proteins, BAX, BAK, BID, BAD, *etc.*, control apoptotic mitochondrial events by regulating mitochondrial membrane permeability [67,75]. Resistance to apoptosis is a hallmark of cancer [76]. Accumulating research findings demonstrated that CE and DE exert anti-cancer effects by inducing apoptosis in different type of cancers. Kim *et al.* showed that DE can dose-dependently induce apoptosis in DU145 human prostate cancer cells by activation of poly (ADP-ribose) polymerase (PARP) and caspases 8, 9, 7, and 3 and increasing the expression of the pro-apoptotic proteins [11]. Choi *et al.* reported that there is a marked increase in the expression of the apoptotic protein BAX and the downstream target p53, causing the release of cytochrome C from the mitochondria, and in turn, triggering the intrinsic signaling pathways of apoptosis in DE-treated SK-OV-3 ovarian cancer cells [9]. Oh *et al.* demonstrated that DE inhibits nuclear transcription factor-κB (NF-κB) activation and enhances caspase-8 and caspase-3 activities to render HL-60 cells susceptible to tumor necrosis factor-α (TNF-α)-induced apoptosis [77]. It is also reported that DE induces apoptosis in human leukemia HL-60 cells by activating caspase-3 after a reduction in mitochondrial membrane potential [78]. In addition, DE inhibits survival signaling through the Janus tyrosine kinase (JAK)-signal transducer and activator of transcription-3 (STAT3) signaling and induces apoptosis in breast cancer MDA-MB-231 cells by up-regulation of BAX and BAD, down-regulation of BCL-2 and BCL-XL, and nuclear relocation of the mitochondrial factors apoptosis-inducing factor and Endo G [23].

Lee *et al.* found that CE induces HL-60 human leukemia cells apoptosis by ROS-mediated mitochondrial permeability transition and cytochrome C release, and this apoptotic pathway associated with the generation of ROS and disruption of mitochondrial membrane potential (Δψ_m_) can be blocked by the antioxidant *N*-acetylcysteine [52]. Similar apoptotic mechanism is also observed in the CE-treated T24 human bladder cancer cells [13] and CE-treated platinum-resistant human ovarian cancer cells [39]. Furthermore, CE can trigger apoptosis by depleting intracellular thiols. Pretreatment with sulfhydryl compounds such as GSH, *N*-acetyl-l-cysteine, dithiothreitol and 2-mercaptoethanol almost completely blocked the CE-induced apoptosis, and overexpression of BCL-2 also significantly attenuated the effects of CE [68]. Further studies show that ROS-mediated c-Jun *N*-Terminal Kinase (JNK) activation plays a key role in CE-induced apoptosis in U937 cells [79]. Kim *et al.* suggested that CE induces apoptosis in 11Z human endometriotic epithelial cells by inhibiting the prosurvival NF-κB and Akt pathway, leading to the down-regulation of anti-apoptotic protein BCL-XL and the activation of caspases [80].

### 4.6. The Death Receptor-Mediated Extrinsic Pathway

The extrinsic signaling pathways initiating apoptosis involve transmembrane receptor-mediated interactions. So far, the best-characterized ligand and corresponding death receptor models include FasL/FasR, TNF-α/TNFR1, Apo3L/DR3, Apo2L/DR4 and Apo2L/DR5 [81,82,83], of which, the first two models can best depict the sequence of events which define the extrinsic phase of apoptosis. The binding of Fas or TNF ligand to Fas or TNF receptor, respectively, will result in formation of a death-inducing signaling complex (DISC), including the TNF receptor-associated death domain (TRADD), Fas-associated death domain (FADD) and Receptor-interacting protein (RIP), in cytoplasm, and the autocatalytic activation of procaspase-8. Once caspase-8 is activated, the execution phase of apoptosis is triggered [84,85]. Choi found that CE induces apoptosis in estrogen receptor-negative breast cancer MDA-MB-231 cells through the extrinsic pathway, characterized as the activation of Fas, caspase-8, caspase-3, and degradation of PARP without disruption of mitochondrial membrane potential and changes in the expression of Bcl-2 and Bax proteins [42]. Moreover, Kanno *et al.* suggested that CE-induced apoptosis in human B cell leukemia NALM-6 cells do not change Fas-associated factor 1 (FAF1), but increase the phosphorylation of Fas-associated death domain (FADD) at serine 194. Moreover, CE treatment can time-dependently activate caspase-8 and -9 and degrade PARP in cells. Pretreatment of cells with caspase-3, -8, and broad-spectrum caspase inhibitors, can significantly block CE-induced apoptosis, but the caspase-9 inhibitor fails to block apoptosis [65].

### 4.7. The ER Stress Pathway

The ER, a central organelle involved in lipid synthesis, protein folding, and maturation, plays a crucial role in the regulation of apoptosis. Toxic insults, such as hypoxia, Ca^2+^ overload, failure of protein synthesis, folding, transport or degradation, can disturb the ER function and result in ER stress, which triggers several specific signaling pathways associated with apoptosis [86,87,88]. Hsu *et al.* found that DE induces apoptosis in liver cancer HepG2 and PLC/PRF/5 cells by increase of Ca^2+^ mobilization and activation of extracellular signal-regulated kinase 1/2 (ERK1/2) and p38, which subsequently causes c-Jun NH2-terminal kinase (JNK) activation and results in AIF and Endo G nuclear relocation, triggering apoptosis [6]. The results of Hung *et al.* demonstrated that DE induced-apoptosis in human non-small cell lung A549 and NCI-H460 cells also follows the ER stress pathway, characterized by changes in cytosol-Ca^2+^ levels, PKR-like ER kinase (PERK) phosphorylation, and caspase-4 activation. The release of Ca^2+^ triggered the production of ROS, which further enhances Ca^2+^ overloading and subsequently activates p38, JNK and ERK1/2 [89].

### 4.8. Anti-Cancer Metastasis and Invasion

Tumor recurrence and metastasis is the main cause of death in cancer patients, and matrix metalloproteinases (MMPs) are assumed to play a major role in changing the tumor microenvironment affecting tumor growth, progression, invasion, metastasis and angiogenesis. Therefore, MMPs have been considered as novel therapeutic targets for cancer treatment [90,91,92,93]. MMP-9 can degrade collagen IV and V of the extracellular matrix (ECM) and gelatin. The change of its expression is closely related to tumor metastasis in various tumors [94]. It was documented that DE inhibits MMP-9 secretion and stimulates TIMP-2 secretion to reduce migration of DU145 and TRAMP-C2 cells [11]. In addition, MMP-2 and MMP-9 levels are significantly reduced in human soft tissue sarcoma TE-671 cells with DE treatment, but significantly increased in SW-982 and TE-671 cells with CE treatment. Interestingly, CE and DE both can significantly reduce the invasion potential of cancer cells. These results indicate that the anti-metastasis mechanism of CE may be different from that of DE [45]. Choi *et al.* investigated the anti-cancer effects of CE and its underlying mechanisms against TNFα-induced breast cancer MDA-MB-231 cells migration and invasion. They found that administration of CE compared to the control, inhibits the growth of tumors and their metastases. The mechanism may be associated with a decrease of P-IKK, P-IKB, and Nuclear p65 in NF-κB signalling pathways [95].

## 5. Reversion of Multidrug Resistance

Drug resistance, including primary drug resistance (PDR) and multiple drug resistance (MDR), is the primary reasons for the failure of cancer chemotherapy in cancer patients. MDR is closely associated with the expression of ATP binding cassette transporters, such as ABCB1/MDR1, ABCC1/MRP1, and ABCG2/BCRP1 [96,97,98,99]. Current research demonstrated that DE inhibits the growth of three kinds of chemo-resistant cancer cells (IC_50_ lower than 10 μg/mL) by reducing the expression of ABCB1/MDR1 and ABCG2/BCRP1, indicating that this compound has a potential to circumvent multidrug resistance in these cells [44]. In addition, it was also reported that CE is more potent than cisplatin in inhibiting cell growth in three platinum-resistant ovarian cancer cell lines (MPSC1^PT^, A2780^PT^, and SKOV3^PT^), moreover, it functions with cisplatin to induce cell death in platinum-resistant ovarian cancer cells [39]. Taken together, these data suggested that CE, as well as DE, alone or in combination with other chemotherapeutic agents, may be of therapeutic potential in chemo-resistant cancer.

## 6. Anti-Angiogenic Activity

Tumor angiogenesis plays a crucial role in tumor growth and metastasis [100,101]. Vascular endothelial growth factor (VEGF) is a most powerful tumor angiogenesis factor, which can interact with its cognate receptors, KDR/Flk-1 and Flt-1 to promote the growth of new blood vessels. Therefore, inhibition of angiogenesis by blocking angiogenesis-related factors has become a potential therapeutic strategy for cancer treatment [102]. Hao *et al.* demonstrated that CE and DE inhibit the growth and survival of human lung cancer A549 cells and the expression of VEGF with a non-toxic concentration [40]. In addition, CE can inhibit angiogenic response *in vitro* and *in vivo* by blocking the, VEGFR KDR/Flk-1 angiogenic factor signaling pathway [103]. Similar results were reported that DE can inhibit human umbilical vein endothelial cell proliferation and capillary-like tube formation *in vitro*, moreover, it also showed an anti-angiogenic effect in the matrigel-plug nude mice model [46]. These studies suggested that CE and DE are potent angiogenesis inhibitors with a potential to be adopted as a novel agents in anticancer therapy.

## 7. Induction of Cancer Cell Differentiation

Most cancer cells persist in a highly proliferative state and thus outgrow their normal cellular counterparts due to their lack of capacity to mature into non-replicating adult cells. Therefore, chemical or biological inducers of terminal differentiation represent an alternative approach to the treatment of cancer [104]. Cell differentiation is closely related to the activation of protein kinase C (PKC) signaling pathways [105,106,107]. Studies from Kim *et al.* demonstrated that CE combined with 1,25-(OH)_2_ Vitamin D_3_ induces HL-60 cells differentiation via activation of a variety of protein kinases including PKC, MAPK and PI3-K and deactivating NF-κB [38]. In addition, Choi *et al.* showed that CE-treated HL-60 cells can develop the characteristics of differentiated cells, and approximately 80% of the CE-treated cells become stainable with nitroblue-tetrazolium, compared to only 13.2% of the untreated cells. The mechanisms may be associated with the increased expression of both membrane antigens CD14 and CD66b [108]. It was reported that CE increase all-trans retinoic acid (ATRA)-induced HL-60 cell differentiation into a granulocytic lineage by inhibiting NF-κB DNA-binding activity. Signaling kinases PKC, ERK, JNK and PI3-K are also involved in the ATRA-induced differentiation enhanced by CE [109]. Therefore, CE can be thought to be a potential inducer of cancer cell differentiation.

## 8. Anti-Tumor Activity, Pharmacokinetics and Metabolism of CE and DE *in Vivo*

The evaluation of anti-tumor activity of drug candidates in animal models is crucial for determining whether they are worth being carried out further in preclinical and clinical studies. Choi *et al.* found that the administration of CE (intraperitoneally once a day for seven days) significantly suppresses tumor growth and increases survival in a 3LL Lewis lung carcinoma-bearing model. Furthermore, there is no weight loss in non-tumor-bearing mice treated with CE (7.5 mg/kg) [79]. It was also reported that the intraperitoneal administration of CE at the dose of 5 or 10 mg/kg/day body weight for 10 days can significantly inhibit the growth of platinum-resistant SKOV3^PT^ as compared with vehicle controls [39]. Results of Kuo and Chio *et al.* indicated that CE or DE significantly inhibits MDA-MB-231 xenograft growth and metastases without causing any side effects to the mice [23,95]. Hsu and Hung *et al.* suggested that DE may be a novel anticancer agent for the treatment of liver cancer and non-small cell lung cancer based on animal studies both revealing a dramatic 50% reduction in tumor volume after 45 and 28 days of treatment, respectively [6,89]. Moreover, Wang *et al.* reported that DE significantly inhibits neo-vascularization in a concentration-dependent manner, demonstrating that it has potential inhibitory activity in growth factors-induced angiogenesis *in vivo* [46]. Recently, Peng *et al.* found that CE combined with DE exerts a synergistic anti-cancer effect on breast cancer MCF-7 xenografts [110].

As described above, numerous preclinical studies indicated that CE and DE are novel anticancer agents for prevention and therapy of cancer. Therefore, studies on potential pharmacokinetics and metabolism are essential for better translating such promising observations into clinic. Within the past several years, to the best of our knowledge, only a few groups have evaluated the pharmacokinetics of CE and DE *in vivo*. A sensitive UPLC-MS/MS for the quantification of CE and DE in biological matrices has been developed by Hu *et al.* and successfully used to analyze the pharmacokinetics of the two compounds [111]. After oral administration of the mix at a single dose at 125 mg/kg mixed solution (containing 25 mg CE and 100 mg DE) to Wistar rats, peak concentrations of CE and DE were 0.024 ± 0.004 and 0.063 ± 0.002 mg/L (*C*_max_) reach 9.0 ± 1.5 and 6.0 ± 1.1 h (*T*_max_), respectively. The half-life (*t*_1/2_) and area under plasma concentration (*AUC*_0–48_) were found to be 4.97 ± 1.07 and 5.44 ± 1.13 h, and 0.33 ± 0.03 and 1.09 ± 0.15 mg/L/h, respectively. Moreover, Zhang *et al.* studied the pharmacokinetic of CE and DE after oral administration of traditional medicine *Aucklandia lappa* Decne by LC/MS/MS. The pharmacokinetic parameters are as follows: *T*_max_ is 10.46 and 12.39 h for CE and DE, respectively. The *t*_1/2_ of CE and DE was calculated to be 5.54 ± 0.81 and 4.32 ± 0.71 h, respectively and the *AUC* of CE and DE was found to be 308.83 and 7884.51 ng·h/mL, respectively [112]. CE and DE are very poorly absorbed in rats after oral administration due to their poor water solubility. Therefore, Peng *et al.* investigated the pharmacokinetics and metabolism of the two compounds in rats after a single intravenous administration [113]. The *C*_max_ of CE and DE were observed to be 12.29 ± 1.47 and 5.79 ± 0.13 μg/mL, respectively, and the *AUC*_(0–∞)_ of CE and DE were calculated to be 3.11 ± 0.13 and 1.37 ± 0.10 μg h/mL, respectively. The *t*_1/2_ of CE and DE are 1.16 ± 0.06 and 2.33 ± 0.58 h, respectively. Further, four metabolites of CE and six metabolites of DE were discovered from the plasma, urine and feces of rats by an ultraperformance liquid chromatography/quadrupole time-of-flight mass spectrometry system (UPLC-Q/TOF MS). In addition, they found that the main metabolic pathway of DE and CE is phase І and II biotransformation, respectively. The information may be useful for the further development of the two drug candidates.

## 9. Conclusions and Future Prospects

This review briefly summarizes the anti-tumor mechanisms and activities of CE and DE. Taken together, these results strengthen the hypothesis that the two compounds could exert multi-targeted chemopreventive effects by blocking different stages of carcinogenesis and progression with a safe pharmacological effect. Although there have been massive studies on anti-cancer activity and associated molecular mechanisms of CE and DE, it is noteworthy that current research lacks systematic evaluation. The targeting proteins of CE and/or DE are not fully clarified, and the number of multicenter, large sample, double-blind, and randomized chemoprevention clinical trials with CE and/or DE are very few. Therefore, their antitumor activities and mechanisms need be further studied. CE and DE usually coexist in medicinal plants, such as *Saussurea lappa* and *Laurus nobilis*, and they have similar chemical properties, moreover, the combination treatment of CE and DE showed a synergistic anti-cancer effect. Therefore, we think combination of the two compounds are likely to be more attractive anticancer agents than CE or DE alone, and simultaneously extraction of the two compounds from their natural sources may be more valuable than chemical synthesis of CE or DE alone.
